# Characterization of a serologically atypical *Shigella flexneri* Z isolated from diarrheal patients in Bangladesh and a proposed serological scheme for *Shigella flexneri*

**DOI:** 10.1371/journal.pone.0202704

**Published:** 2018-08-24

**Authors:** Mohammad Shahnaij, Hasan A. Latif, Ishrat J. Azmi, Mohammed Badrul Amin, Sharmin J. Luna, Mohammad Aminul Islam, Kaisar Ali Talukder

**Affiliations:** 1 Laboratory Sciences and Services Division, International Centre for Diarrhoeal Disease Research, Bangladesh, Dhaka, Bangladesh; 2 Department of Biotechnology and Genetic Engineering, Mawlana Bhashani Science and Technology University, Santosh, Tangail, Bangladesh; Defense Threat Reduction Agency, UNITED STATES

## Abstract

**Background:**

Atypical *Shigella flexneri* Z variant, that agglutinate with E1037 group factor specific monoclonal antisera against *Shigella flexneri* IV-I but not with other group or type specific antisera, has continuously being isolated in Bangladesh since 1997. Later this serotype has been reported in Indonesia, China and Argentina. Despite being a provisional serotype, continuous isolation of these strains in diverse geographical regions implicated a great necessity to study the overall characteristics of these strains. Therefore, we extensively characterized *S*. *flexneri* Z strains using various phenotypic and molecular tools.

**Method:**

Of 3569 *S*. *flexneri* isolated between 1997 and 2015, 95 strains were identified as *S*. *flexneri* Z using a panel of polyvalent absorbed antisera and monoclonal antisera of *S*. *flexneri* (MASF). Of them, randomly selected 65 strains were molecular O-serotyped using multiplex PCR and characterized using different phenotypic and molecular techniques (i.e.biotyping, plasmid profile, virulence marker and PFGE) to determine relationship with other subserotypes of *S*. *flexneri*.

**Results:**

All these atypical *S*. *flexneri* Z strains were agglutinated with MASF B and IV-I antisera. Concordantly, these strains were positive to opt-gene, responsible for MASF IV-I sero-positive phenotype. However, molecular O-serotyping of all 65 strains could not differentiate between Z and Yb giving similar amplification products (*wzx1-5* and *opt*). Contrarily, MASF based serotypic scheme distinguished among Z and Yb as well as Ya. All these *S*. *flexneri* Z showed typical biochemical reaction of *S*. *flexneri*, harboured a 140 MDa virulence plasmid and virulence markers namely *ipaH*, *ial*, *sen*, *sigA* and *sepA* genes. Along with the virulence plasmid, small plasmids (2.6, 1.8 and 1.6 MDa) were present as core plasmid. Moreover, a middle ranged plasmid and a 4.0 MDa sized plasmid were observed in 65% and 20% strains, respectively. Analysis of PFGE on *Xba*I-digested chromosomal DNA of Bangladeshi strains showed that *S*. *flexneri* Z had a close relatedness with Ya and Yb but completely different than the strains of Xa, Xb, 2a and 2b. This observation was found to be unequivocal while the overall result of biotyping, plasmid profile, and virulence factors was compared. Therefore, we conclude that these atypical serotype Z isolated in Bangladesh had a clonal relationship with Ya and Yb of Bangladesh and the *opt* gene played an important role in serotypic switching among them. Current serotyping scheme of *S*. *flexneri* strains fails to place many such atypical strains (1c, 1c+6, 1d, type 4, and 4c) including *S*. *flexneri* Z isolated from different parts of the world. Therefore, an updated serotyping scheme for identification of subserotypes of *S*. *flexneri* has been proposed to avoid multiple naming of the same subserotype having similar agglutination pattern.

## Introduction

Shigellosis is one of the major causes of morbidity and mortality in developing countries like Bangladesh, especially among children under five years [1–3]. A shigellosis survey between 2000 and 2004 in six Asian countries including Bangladesh, China, Pakistan, Indonesia, Vietnam and Thailand showed that the overall annual incidence of treated shigellosis was 2.1/1000 per year in all ages and 13.2/1000 per year in children under 5 years old, which was much higher than that in industrialized countries [2]. The Global Burden of Disease Study 2015 estimates that Shigellosis causes 164,410 deaths worldwide in which 98.5% occurred in low and middle income countries and 33% in children under 5 years old [4]. The disease is caused by *Shigella* spp. that colonizes the intestinal mucosa and continues to threaten public health mainly in less developed countries with conditions of poor sanitation. Moreover, clinical infection can be carried out by as little as 10 *Shigella* organisms even without neutralization of gastric acid [5]. Despite a significant global burden of the disease and worldwide spread of antibiotics resistance, no effective vaccine against shigellosis is widely available except for a few candidate vaccines that are under development [6]. The genetic and epidemiological switching leading to chronologic variability between serotypes, and emergence of atypical and new variants strains throughout the world often complicates the development of an effective vaccine[7–11]. As immunity to *Shigella* is serotype specific, vaccine protection will therefore depend on the representation of each serotype in the vaccine or at least the predominant serotypes. Therefore, estimating the disease burden due to *Shigella* infection and understanding it’s epidemiology in countries where the disease is mostly prevalent have utmost importance[12–14].

Based on biochemical and serological properties, the genus *Shigella* is divided into four species: *S*. *dysenteriae*, *S*. *flexneri*, *S*. *sonnei*, and *S*.*boydii*. *S*. *flexneri* causes far more cases of dysentery than other species of *Shigella* in developing countries including Bangladesh [9, 15]. A set of absorbed rabbit antisera against the type specific (I-VI) antigenic factors and group specific (3.4, 6, 7.8) factors are routinely being used for serotyping of *S*. *flexneri* that subdivide isolates into 13 serotypes (1a, 1b, 2a, 2b, 3a, 3b, 4a, 4b, 5a, 5b, X, Y and 6) [16, 17]. However, this serological scheme of *S*. *flexneri* is not comprehensive since this cannot type several atypical and novel subserotypes [10, 18]. In addition, the absorbed antisera often show residual cross-reactivity or are of lower titer since there are vast structural similarities between different serotypes of *S*. *flexneri* [19]. To overcome this constrain, monoclonal antisera of *S*. *flexneri* (MASF) can be used instead of the commercially available absorbed antisera [20]. Until recent, six type specific (I, II, IV-2, V, VI and 1c) and four group specific (Y-5, 6, 7.8 and IV-I) monoclonal antibodies are being developed in order to type all the existing subserotypes of *S*. *flexneri* including nontypeable strains [16, 17].

A Wzy/Wzx dependent pathway, common to all *S*. *flexneri* except for *S*. *flexneri* type 6, synthesize the common repeating tetrasaccharide unit (referred to serotype Y), to which type and/or group specific determinants including a-D-glucopyranosyl and/or O-acetyl and/or phosphoethanol amine (PEtN) groups are added exhibiting variation in O antigen [21–23]. These modifications are mostly conferred by prophage-encoded glucosyltransferase and/or acetyltransferase genes [24–26]. Recently a plasmid (6.8 Kb) mediated *opt* gene conferring the synthesis of a group antigen E1037 has been reported [22], albeit the antigen was first identified in 1984 in a provisional strain E1037 of *S*. *flexneri* by Berhard Rows [27]. Later, the antigen was detected in some strains of serotypes 4a, X, Y and 6 using E1037 specific antiserum, MASF IV-I [20]. Immunogenetical studies showed that the *opt*O gene encoding the phosphoethanol amine transferase enzyme that transfers ethanolamine phosphate group predominantly to Rha^II^ and rarely to both Rha^II^ and/or Rha^III^ residues of O antigen [28]. Until recently eight serotype converting genes (*gtr*I, *gtr*II, *gtr*IV, *gtr*V, *oac*, *gtr*X, *opt* and *gtr*Ic) responsible for the transfer of α-D-glucopyranosyl, O-acetyl and phosphoethanol amine (PEtN) have been identified [24, 29]. Based on the presence of these genes, PCR based O-serotyping technique was developed for the rapid detection of serotypes of *S*. *flexneri* [29, 30].

Isolation of uncommon serotypes and subserotypes of *Shigella* spp. particularly of *S*. *flexneri* is not a rare event. Atypical strains or novel subserotypes (i.e. *S*. *flexneri* 1d, type 4, 7a and 7b etc.) are being isolated in different parts of the world including Bangladesh. Even several atypical serotypes became predominant over the established serotypes in developing countries [9, 10, 18]. For example, in China, the predominant serotype of *S*. *flexneri* 2a was replaced by atypical strain of *S*. *flexneri* (-:7.8, E1037) in 2010 [31]. Despite their epidemiological importance, these serotypes are not included in the current serological scheme. Use of monoclonal antisera may provide an extended serological scheme covering these atypical strains which remain in provisional status [18, 32–34]. Among the provisional strains of *S*. *flexneri*, a group of atypical strains that agglutinate only with MASF IV-1 but with any type or group antigen-specific antisera tested have been reported earlier (previously reported as 4X) and have continuously being isolated in Bangladesh since 1997 [10]. Later, this serologically atypical strain (as 4X) has also been reported from Indonesia and Argentina in 2002 and in 2010, respectively. Recently, isolates with the same serological characteristics have been reported (provisionally designated as serotype 4s) form China [35–37]. We renamed this atypical strain as *S*. *flexneri* Z for the systemic nomenclature of *Shigella flexneri*. This study was aimed to characterize the Bangladeshi strains of *S*. *flexneri* Z using different phenotypic and molecular techniques. In addition, we proposed an updated MASF based serotyping scheme for the identification of all typical and atypical serotypes and sub-serotypes of *S*. *flexneri*.

## Materials and methods

### Ethics statement

This study was exempted from institutional review board (IRB) approval at the International Centre for Diarrhoeal Disease Research, Bangladesh (icddr,b) given that no experimental procedures were performed and no patient or subject identifiers were collected.

### Bacterial strains

A total 3569 of *S*. *flexneri* strains were isolated and identified from patients with diarrhoea attending the Dhaka treatment center of the International Centre for Diarrhoeal Disease Research, Bangladesh (icddr,b) following the standard microbiological and biochemical methods [38], between January 1997 and December 2015. In this study, randomly selected 65 strains of *S*. *flexneri* Z [-:E1037] variants were included for characterization using different phenotypic and genotypic techniques and compared the same with other subserotypes of *S*. *flexneri* obtained from our laboratory collection. All strains were grown in trypticase soy broth with 0.3% yeast extract (TSBY) and stored at -70°C after adding 15% glycerol. YSH6000 *S*. *flexneri* 2a, having 140MDa invasive plasmid, were used as positive control in the polymerase chain reaction (PCR) for detection of *ipaH* gene, *ial*, *Shigella* enterotoxin gene (*set*) and Serine protease autotransporter of Enterobacteriaceae (SPATE) genes (*sig*A, *pic*, *sat* and *sep*A) while an *E*. *coli* (ATCC 25922) strain lacking 140 MDa plasmid serves as a negative controls in PCR and also antibiotic susceptibility testing.

### Serotyping

Serotyping of these isolates was performed using (i) a commercially available antisera kit (Denka Seiken, Tokyo, Japan) specific for all type- and group-factor antigens and (ii) a panel of monoclonal antisera (Reagensia AB, Stockholm, Sweden) reagents specific for all *S*. *flexneri* type- and group-factor antigens [10]. For the serotypic designation, we followed an extended version of serotyping scheme described in Table 1. PCR based assay targeting the O-antigen synthesis gene *wzx* and the O-antigen modification genes: *gtr*I, *gtr*IC, *gtr*II, oac, *gtr*IV, *gtr*V, *gtr*X, and *opt* were used to detect the molecular serotype of *S*. *flexneri* according to procedure described elsewhere [29, 30].

**Table 1:** Proposed serotyping scheme for the nomenclature of *Shigella flexneri* using a panel of monoclonal antisera(MASF).

Serotype	Previously designated name	Type antigen specific antisera (MASF)						MASF	Group antigen specific antisera (MASF)			
		I	II	IV-2	V	VI	Ic	B	Y-5	6	7,8	IV-I
*S*. *flexneri* 1a	1a	+						+	+			
*S*. *flexneri* 1b	1b	+						+		+		
*S*. *flexneri* 1d	1d[18]	+						+			+	
*S*. *flexneri* 2a	2a		+					+	+			
*S*. *flexneri* 2b	2b		+					+			+	
*S*. *flexneri* 3a	3a							+		+	+	
*S*. *flexneri* 3b	3b							+		+		
*S*. *flexneri* 4a	4a			+				+	+			
*S*. *flexneri* 4b	4b			+				+		+		
*S*. *flexneri* 4c	4c [17]			+				+			+	
*S*. *flexneri* 4d	type4 [33]			+				+				+
*S*. *flexneri* 4e	4a, 4av [39]			+				+	+			+
*S*. *flexneri* 5a	5a				+			+	+			
*S*. *flexneri* 5b	5b				+			+			+	
*S*. *flexneri* 6a	type 6					+		+				
*S*. *flexneri* 6b	type 6					+		+				+
*S*. *flexneri* 7a	1c						+	+				
*S*. *flexneri* 7b	1c+6 [30]						+	+		+		
*S*. *flexneri* Xa	X							+			+	
*S*. *flexneri* Xb	Xv [22]							+			+	+
*S*. *flexneri* Ya	Y							+	+			
*S*. *flexneri* Yb	Yv [22]							+	+			+
*S*. *flexneri* Z	4X, 4s [10, 35]							+				+

### Biochemical characterization

The biochemical reactions of the strains were performed by standard biochemical methods [38].

### Antimicrobial susceptibility testing

Bacterial susceptibility to antimicrobial agents including ampicillin (AMP 10 μg), sulfomethoxazole-trimethoprim (SXT 23.75/1.25 μg), nalidixic acid (NAL 30 μg), ciprofloxacin (CIP 5 μg), ceftriaxone (CRO 30 μg), ceftazidime (CAZ 30 μg) and amoxicillin-clavulanic acid (AMC 20/10 μg) available from commercial manufacturer (Oxoid, Basingstoke, United Kingdom) were determined by the disk diffusion method as recommended by the Clinical and Laboratory Standards Institute (CLSI, 2017). *E*. *coli* ATCC 25922, *E*. *coli* ATCC 35218 and *Staphylococcus aureus* ATCC 25923 were used as control strains for susceptibility testing.

### Isolation of plasmid DNA

Plasmid DNA was prepared according to the alkaline lysis method of Kado and Liu with some modifications described elsewhere [33, 40]. The molecular weight of the unknown plasmid DNA was assessed by comparing with the mobility of the known molecular weight plasmids [41]. Plasmids present in strains *E*. *coli* PDK- 9 (140, 105, 2.7 and 2.1 MDa), R1 (62 MDa), RP-4 (36 MDa), Sa (23 MDa) and V517 (35.8, 4.8, 3.7, 3.4, 3.1, 2.0, 1.8 and 1.4 MDa) were used as molecular mass standards.

### Determination of the role of transferable plasmid factor

Three strains of *S*. *flexneri* Z, K-5851 (AMP^R^SXT^R^NAL^S^), KD-1170 (AMP^R^SXT^R^NAL^S^CAZ^R^CRO^R^AMC^R^), and K-7468 (AMP^R^SXT^R^NAL^S^CAZ^R^CRO^R^AMC^R^) were selected as donor strain for the conjugation experiment. Each of the selected donor strains were mated with a recipient strain *E*. *coli* K-12 (lac^+^NAL^R^F^-^) according to the method described previously [33]. Transconjugants colonies were selected on the MacConkey agar plates containing ampicilin (100 mg/L) and nalidixic acid (50 mg/L) and the transfer frequency of the resistance plasmid was calculated by a method described earlier [42]. Plasmid profile analysis, plasmid curing and antimicrobial susceptibility test of the transconjugants strains were performed to determine the transfer of plasmids with antibiotic resistance phenotype.

### Detection of virulence genes and SPATE genes by PCR

All the strains of 2a, 2b, Xa, Xb, Ya (n = 25), Yb (n = 10) and Z (n = 65) were tested for virulence genes: *set*1 (ShET-1), *sen* (ShET-2), *ial*, *ipa*H genes, and a set of SPATE genes *(sat*, *pic*, *sepA and sigA)* commonly found in *Shigella* spp. by PCR using primer sets described previously with mentioned annealing temperature [43, 44].

### Pulsed-field gel electrophoresis (PFGE)

To determine the clonal relationship of *S*. *flexneri* Z variant and closely related subserotypes, 29 representative strains of *S*. *flexneri* Z were compared with 13 strains of *S*. *flexneri* Ya, three *S*. *flexneri* Yb, three *S*. *flexneri* Xa and one strains of Xb using PFGE typing. Intact agarose embedded chromosomal DNA was prepared according to the guideline of pulsenet [45, 46]. Genomic DNA was digested with the *Xba*I restriction enzyme for 4 h at 37°C and the restriction fragments were separated by using CHEF-MAPPER system apparatus (Bio-Rad Laboratories) under the following conditions: switching time from 5 s to 35 s at 6 V cm^−1^ for 18 h at 14°C. PFGE images were analyzed using the fingerprint analysis software BioNumerics version 4.5 (Applied Maths; Kortrijk, Belgium).The dendrogram constructed using the PFGE patterns was generated by the UPGMA algorithm with the Dice-predicted similarity value of two patterns at 1.0% pattern optimization and 1.5% band position tolerance.

## Results

### Serological typing

All the isolates of *S*. *flexneri* were confirmed using the MASF B antisera in slide agglutination reaction suggesting that these strains were subserotypes of *S*. *flexneri*. Among 3569 *S*. *flexneri*, 95 isolates (2.7%) were identified as the atypical strains of *S*. *flexneri*, designated as *S*. *flexneri* Z that strongly agglutinated only with MASF IV-I but did not agglutinate with other group or type specific antisera of commercially available both absorbed polyvalent and monoclonal antisera. These 95 strains of *S*. *flexneri* Z neither agglutinated with type specific antisera nor with group specific antisera available in commercial antisera kit. The isolation rate of *S*. *flexneri* Z was reduced from 9.7% in 1997–2000 to 1.1% in 2013–15 (Table 2).

**Table 2:** Prevalence of *Shigella flexneri* in Bangladesh between 1997 and 2015.

	1997–00	2001–04	2005–08	2009–12	2013–15	1997–2015
*S*. *flexneri* 1a	2	6	5	1	1	15
*S*. *flexneri* 1b	69	73	103	22	5	272
*S*. *flexneri* 1d	0	0	0	0	0	0
*S*. *flexneri* 2a	70	713	234	382	203	1602
*S*. *flexneri* 2b	105	217	59	20	3	404
*S*. *flexneri* 3a	70	263	111	100	46	590
*S*. *flexneri* 3b	0	1	1	4	1	7
*S*. *flexneri* 4a	0	2	0	2	0	4
*S*. *flexneri* 4b	0	1	0	1	0	2
*S*. *flexneri* 4c	0	0	0	0	0	0
*S*. *flexneri* 4d	17	32	16	9	11	85
*S*. *flexneri* 4e	0	0	0	1	0	1
*S*. *flexneri* 5a	1	0	3	1	0	5
*S*. *flexneri* 5b	0	0	1	0	0	1
*S*. *flexneri* 6a	12	58	38	68	22	198
*S*. *flexneri* 6b	10	23	16	35	13	97
*S*. *flexneri* 7a	37	80	13	79	37	246
*S*. *flexneri* 7b	0	0	0	0	0	0
*S*. *flexneri* Xa	1	1	2	1	1	6
*S*. *flexneri* Xb	0	0	0	1	0	1
*S*. *flexneri* Ya	10	7	1	8	2	28
*S*. *flexneri* Yb	9	10	0	4	2	25
*S*. *flexneri* Z	39	30	17	5	4	95
Total	404	1477	603	734	351	3569

### Molecular serotyping

Of the 95 *S*. *flexneri* Z isolates, 65 were randomly selected for molecular serotyping using multiplex PCR. All the 65 isolates were positive for wzx_1-5_ gene which is commonly found in all *S*. *flexneri*, except for serotype 6. Additionally, *opt* gene was detected in all the *S*. *flexneri* Z isolates. We detected both *wzx*_1-5_ and *opt* genes in all strains of *S*. *flexneri* Z and Yb.

### Biochemical characterization

All *S*. *flexneri* serotype Z isolates possessed the biochemical characteristics typical of *S*. *flexneri*, including negative reactions in utilization of sodium acetate, rhamnose, xylose, raffinose, ornithine, arginine, and lysine [47]. Around 58% of the isolates showed positive reaction in arabinose fermentation while 70% showed positive reaction in trehalose fermentation at variable time intervals. Interestingly, all the *S*. *flexneri* Z, Ya and Yb isolates produced indole after utilizing tryptophan, which was in contrast to the strains of *S*. *flexneri* 2a, 2b, Xa and Xb. Based on the biochemical reaction patterns *S*. *flexneri* Z isolates appeared to be more similar to *S*. *flexneri* Ya and Yb (Table 3).

**Table 3:** Biochemical characteristics of representative strains of *S*. *flexneri* serotype Z and other serotypes.

Subserotypes of *S*. *flexneri*	No of tested strains	Indole prod^n^	Mannitol	Arginine decarboxylase	Glucose	Arabinose	Raffinose	Rhamnose	Trehalose	Sodium acetate	Maltose	Xylose	Mannose	Biotype
Z	65	100%	100%	4.5%	100%	(58%+)^ǂ^	0%	0%	30% (70%+)	0%	100%	0%	100%	B1
Yb	10	100%	100%	0%	100%	(60%+)	0%	0%	20% (80%+)	0%	100%	0%	100%	B1
Ya	25	100%	100%	4%	100%	(60%)	0%	0%	33% (67%+)	0%	100%	0%	100%	B1
Xa	4	0%	100%	0%	100%	100%	0%	0%	(100%+)	0%	100%	0%	100%	B2
Xb	1	0%	100%	0%	100%	100%	0%	0%	(100%+)	0%	100%	0%	100%	B2
2a	5	0%	100%	0%	100%	80% (20%+)	0%	0%	(100%+)	0%	100%	0%	100%	B2
2b	5	0%	100%	0%	100%	100%	0%	0%	(100%+)	0%	100%	0%	100%	B2

^ǂ^ in parenthesis, strains required prolong incubation time (≥4 days) for fermentation.

### Antibiotic susceptibility test

Of the 65 *S*. *flexneri* Z isolates tested for antibiotic susceptibility, 61% were resistant to amplicillin, followed by 57% of SXT, 55% to nalidixic acid and 41% to ciprofloxacin. Around 45% of isolates were resistant to three or more classes of antibiotics and thus identified these as multi drug resistant (MDR). Two isolates (K-7468 and KD-1170) were resistant to third generation cephalosporin including ceftriaxone, ceftazidime and amoxicillin-clavulanic acid. Both isolates were positive for extended spectrum beta lactamase (ESBL) as detected by using double disc synergy method. None of the isolates were resistant to nalidixic acid and ciprofloxacin.

## Plasmid profile analysis

**Fig 1:**
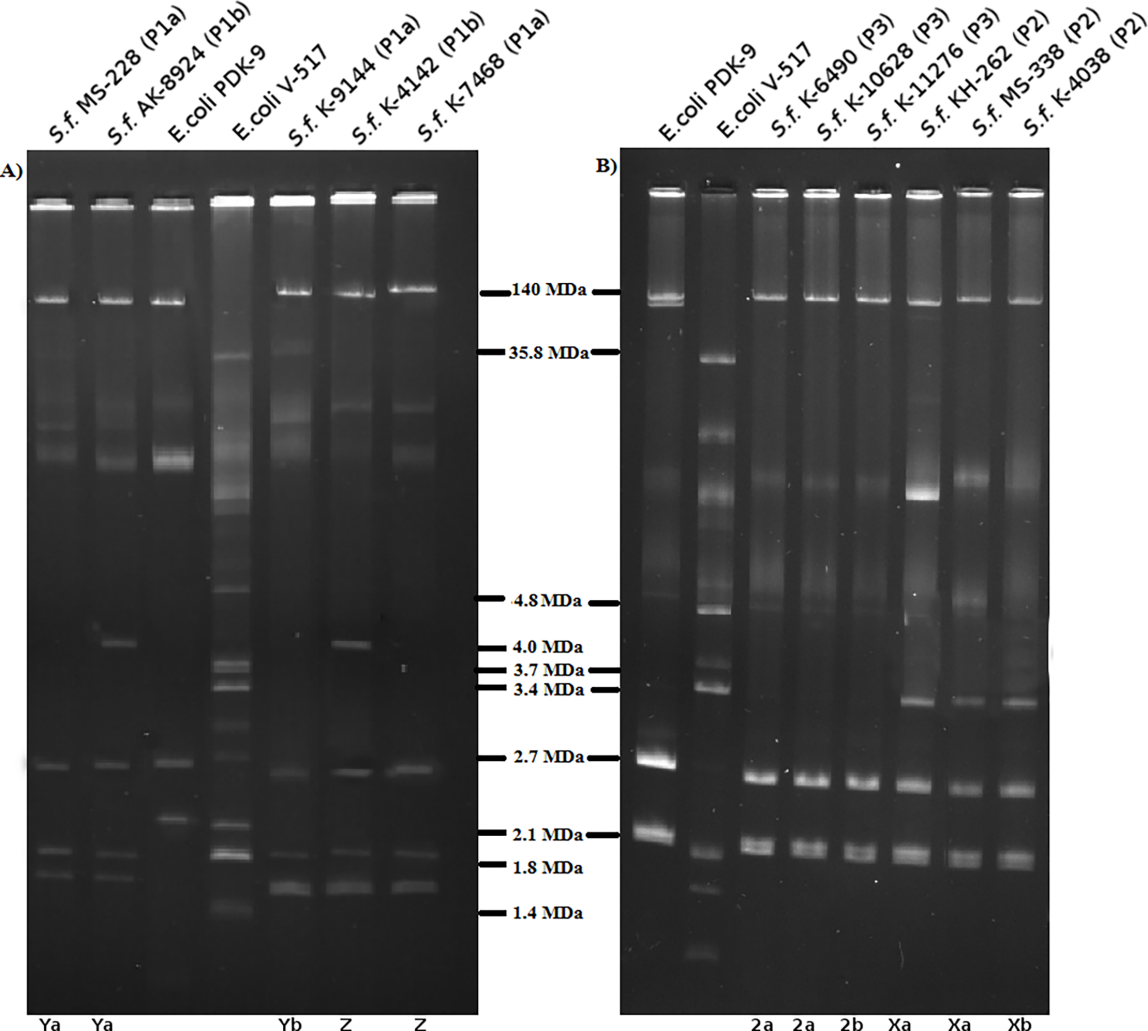
Agarose gel electrophoresis of plasmid DNA showing representative patterns of S. flexneri Z and other subserotypes compared. Agarose gel electrophoresis pattern of plasmid DNA isolated A) from subserotype Ya, Yb and Z, and B) from subserotype 2a, 2b, Xa, and Xb.

Analysis of plasmid DNA revealed that all the strains of *S*. *flexneri* Z harbored the 140 MDa plasmid along with three or four small sized (<6 MDa) plasmids (Fig 1). Additionally, a middle ranged plasmid (20–80 MDa) was detected in 37 strains (62%) of Z variant. The prevalent plasmid pattern P1a (140, 2.6, 1.8 and 1.6 MDa) was found in 39 (87%) strains while the remaining four strains were belonged to P1b (140, 4.0, 2.6, 1.8 and 1.6 MDa). Both plasmid patterns were observed in serotype Ya and Yb (Table 4). However, serotype Xa and Xb contained plasmid pattern P2 (140, 3.4 2.7 and 2.1 MDa) while serotype 2a and 2b had plasmids of slightly different sizes (140, 2.7 and 2.1 MDa) (P3).

**Table 4:** Plasmid profile of representative strains of *S*. *flexneri* serotype Z and other serotypes.

Serotype	No. of strains	140 MDa	80–20 MDa (% +)	4.0 MDa	3.4 MDa	2.7 MDa	2.6 MDa	2.1 MDa	1.8 MDa	1.6 MDa	plasmid pattern
Z	52	+	50%				+		+	+	p1a
Z	13	+	70%	+			+		+	+	p1b
Yb	10	+	100%				+		+	+	p1a
Ya	20	+	75%				+		+	+	p1a
Ya	5	+	23%	+			+		+	+	p1b
Xa	4	+	0%		+	+		+			p2
Xb	1	+	0%		+	+		+			p2
2b	5	+	60%			+		+			p3
2a	5	+	80%			+		+			p3

### Determination of the role of transferable plasmid

Three strains of *S*. *flexneri* Z with different plasmid patterns were selected for conjugation experiment with *E*. *coli* strain K-12 (Lac^+^F^-^NAL^R^). After conjugation, a plasmid of 36 MDa from both K-7468 and KD-1170 strains was transferred to the recipient strain of *E*. *coli* K12 with the full spectrum of resistance to AMP, SXT, CRO, CAZ and AMC. Transfer frequency for both isolates were similar. However, in case of K-5851, a plasmid of 62 MDa was transferred with a frequency of 10-fold higher (6×10−^4^) than the previous two isolates with resistance to AMP and SXT only (Table 5). Transfer of antimicrobial resistance through transmissible plasmids was confirmed by curing the plasmids of transconjugants.

**Table 5:** Transfer of resistance plasmid to E. coli K-12 in conjugation experiment.

Donor	Plasmid pattern (MDa)	Acquired plasmid by strain *E*. *coli* K-12	Transferred resistant phenotype	Transfer frequency of R-plasmid
K-7468	140,36, 2.6, 1.8, 1.6	36	AMP^R^, SXT^R^, CRO^R^, AMC^R^,CAZ^R^	3×10−^5^
KD-1170	140,36, 2.6, 1.8, 1.6	36	AMP^R^, SXT^R^, CRO^R^, AMC^R^,CAZ^R^	5×10−^5^
K-5851	140, 62, 4.0, 2.6, 1.8, 1.6	62	AMP^R^, SXT^R^,	6×10−^4^

^R^Resistant to antimicrobial drug

### Detection of virulence and SPATE genes by PCR

*ipa*H, *ial*, *sen*, *sig*A and *sep*A genes were detected in all the tested *S*. *flexneri* Ya, Yb and Z strains, but none of the isolates were found to be positive for *shet*1, *sat*, *pic* and *pet* genes. However, all these nine genes were detected in each of the strains of serotype Xa, Xb, 2a and 2b.

### Pulsed-field gel electrophoresis

**Fig 2:**
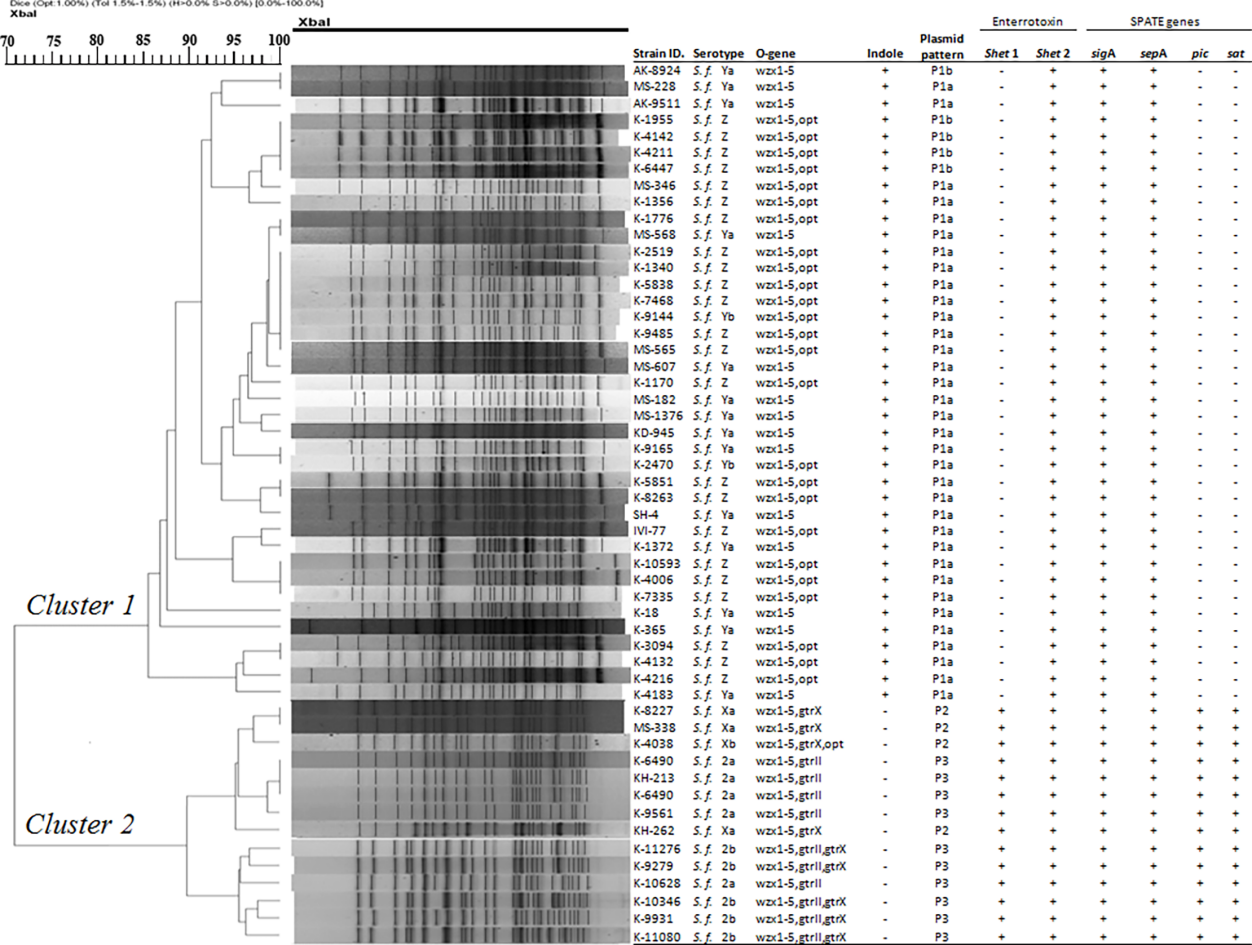
PFGE based phylogenetic relationship of *S*. *flexneri* Z with other serotypes.

The dendrogram generated based on *Xba*1-digested PFGE banding patterns demonstrated that all the 29 strains of *S*. *flexneri* Z isolated from Bangladesh were formed a single cluster (cluster 1) at 80% similarity level which was further divided into four subclusters (1A, 1B, 1C and 1D at 85%). Strains in each of the subclusters were grouped and intermingled with strains of Ya and Yb serotypes. On the other hand, serotypes 2a, 2b, Xa and Xb grouped in cluster 2 were completely different from the cluster 1 (Fig 2).

## Discussion

Diversity of O antigen in *S*. *flexneri* evolves due to presence of serotype converting genes on horizontal transmissible elements [24, 29]. These genes encode enzymes which modify the common tetrasaccharide unit (except serotype 6) of *S*. *flexneri*. As a result, atypical strains or newer subserotypes of *S*. *flexneri* are being isolated from different parts of the world, including Bangladesh [9, 10, 18]. In 2001, we reported three atypical strains of *S*. *flexneri* namely type 4, 1c and 4X which could not be definitively serotyped using commercially available antisera [10]. Therefore, for the identification of subserotypes of *S*. *flexneri*, a set of MASF antisera were used for a definitive serotyping of *S*. *flexneri* that are not included in the current serotyping scheme of *S*. *flexneri*. A group of atypical strains, designated as *S*. *flexneri* Z (previously reported as 4X), that only agglutinated with MASF IV-I but not with any other type or group specific monoclonal antibodies, were isolated in Bangladesh [10]. These atypical strains were also reported from Indonesia and Argentina but not characterized in detail [2, 36]. In 2010, this serotype (provisionally designated as 4s) was reported from China having clonal relationship with serotype 2, Xa, Xb, Ya and Yb [35, 37]. Since characterization of the atypical serotype isolated in Bangladesh remained undone, in this study, a comparative study was carried out to determine their relationship with other serotype of *S*. *flexneri* using different typing tools based on phenotypic and molecular traits.

*S*. *flexneri* Z isolates could not be serotyped using absorbed antisera that are commercially available (Denka Seiken co. Japan), but only agglutinated with MASF IV-I group factor (Table 1). The *opt* gene, responsible for MASF IV-I sero reactive phenotype was detected in all the *S*. *flexneri* Z tested. Although PCR based detection of O-serotype of *S*. *flexneri* and serotyping using monoclonal antisera had showed consistent result, but it could not differentiate between the Yb and Z serotypes that gave similar amplification result (wzx1-5, *opt*). On the other hand, antisera based serotyping could differentiate between these two serotype Yb and Z, since MASF Y-5 specifically agglutinated with Yb strains, but not with Z strains. Since no genetic determinants for Y-5 is available, molecular serotyping of these isolates was not possible. Another limitation of the PCR based serotyping is that due to presence of mutation in O-antigen genes, this method may give discrepant results when cross checked with antisera based serotyping [48]. Moreover, for establishing a novel serotypes, producing and verifying a new antisera for the phenotypic schemes are much easier than identifying and validating a new primer pairs of the atypical strain[48]. Therefore, antisera based serotyping scheme is by far the gold standard for serotyping of *S*. *flexneri* [48].

It has been reported that a plasmid (6.85-10Kb)-borne *opt* gene may transfer to other serotypes via conjugation and eventually can convert their hosts into a MASF IV-I reactive phenotype [37]. Interestingly, we found a similar sized plasmid (~6.4 Kb) in 25% (13/52) strains of *S*. *flexneri* Z. However, a large proportion (75%) of the isolates was negative for this plasmid indicating that other mechanism might be involved in the acquisition and/or transmission of the gene, which accentuates further genetic investigation. Acquisition and transfer of plasmids carrying antibiotic resistance occur quite frequently in *Shigella* spp We found a plasmid of 62 MDa in size conferring resistance to ampicilin and SXT with a high degree of transfer frequency (6×10^−4^). Interestingly, two strains of *S*. *flexneri* Z harbored a 36 MDa transmissible plasmid carrying resistance to 3^rd^ generation cephalosporin (ceftriaxone and ceftazidime) along with AMP and SXT, though the transfer frequency was 10-fold lower than that of the 62 MDa plasmid. Emergence of multiple drug resistance genes and their dissemination among these bacteria complicates the treatment strategy. Hence, more emphasis should be given to new/atypical isolates of *Shigella* spp.

In addition to the transferrable plasmids, all strains of *S*. *flexneri* Z harboured a 140 MDa plasmid along with three small sized stable plasmids (2.6, 1.8 and 1.6 MDa). Although the invasive plasmid of *S*. *flexneri* (140MDa) is extensively studied, little is known about the function of the small plasmid which constitute as a stable DNA pool. Therefore, these stable plasmids can be used as a molecular typing tool for the characterization of *S*. *flexneri* [41]. Overall analysis of plasmid DNA indicated that plasmid patterns of *S*. *flexneri* serotype Z isolates were indistinguishable from that of the serotypes Ya and Yb but distinct from those of serotype X and serotype 2. This observation was also true for biochemical properties, distribution of virulence genes and PFGE banding patterns of the isolates belonged to respective subserotypes and serogroups (Fig 2). The presence of similar plasmid pattern in isolates of different serotypes which are biochemically and genetically related to each other suggests that plasmid profiles may be a useful tool for determining their clonal relatedness.

All strains of serotype Z had biochemical characteristics typical of *S*. *flexneri* and very similar to that of serotypes Ya and Yb. Unlike serotype 2a, 2b, Xa and Xb, all strains of Z as well as Ya and Yb produced indole from tryptophan (Table 3). Although little is known about the effect of tryptophan utilization and indole production on characteristics of *Shigella* spp. it can be used as a biomarker for detection of *S*. *flexneri* Z. The dendrogram based on PFGE banding pattern was a better fit with indole production properties in which all strains belonged to cluster1 (Ya,Yb and Z) produced indole whereas strains of cluster 2 (2a, 2b, Xa and Xb) did not. Furthermore, strains in cluster 1contained relatively less virulence factors (*ipa*H, *ial*, *sen*, *sig*A and *sep*A genes), compared to the strains in cluster 2 (*ipa*H, *ial*, *set*1, *sen*, *sat*, *pic*, *sepA and sigA* genes).

In summary, Bangladeshi isolates of *S*. *flexneri* Ya, Yb and Z were different from isolates of serotypes 2a, 2b, Xa and Xb both at phenotypic and genotypic levels (Fig 2). Although Chinese isolates of the serotype 4s (= serotype Z) have the similar sero-agglutination pattern like S *flexneri* Z, Chinese strains showed clonal relationship with serotype 2, Xa, Xb, Ya and Yb [35–37]. This characteristic differences between isolates from two different regions explained that Bangladeshi isolates of *S*. *flexneri* Z may be emerged from a different clonal population than that of the Chinese isolates. This may be attributed to the insertion of gene carrying MASFIV-1 sero-property into serotype Ya of Bangladeshi isolates through horizontal gene transfer mechanism.

*S*. *flexneri* Z and a few other atypical *S*. *flexneri* have been reported from different parts of the world that require further serological classification [9, 10, 17, 18, 22, 30, 35]. In order to meet this growing demand, we proposed an updated serotyping scheme for the identification of subserotypes of *S*. *flexneri*. Using this MASF based serological scheme it is possible to identify all the reported serotypes and subserotypes of *S*. *flexneri* including the atypical ones (Table 1).

## References

[1] Kotloff KL, Winickoff JP, Ivanoff B, Clemens JD, Swerdlow DL, Sansonetti PJ, et al Global burden of Shigella infections: implications for vaccine development and implementation of control strategies. Bull World Health Organ. 1999;77(8):651–66. Epub 1999/10/12. PubMed Central PMCID: PMCPMC2557719 1997 on Shigella infection. The purpose of the review is to provide data on the global morbidity and mortality caused by the infection and to plan strategies of prevention and treatment. The data obtained from this literature were used to calculate the number of Shigella infection cases and the associated mortality occurring worldwide each year, by age and by clinical category. The burden of Shigella infection was also estimated by serogroup and serotype. A sensitivity analysis was performed to estimate the high and the low range of morbid and fatal cases in each category (mild cases remaining at home, moderate cases requiring outpatient care and severe cases demanding hospitalization). The result of the calculations and analysis revealed that the annual number of Shigella infections throughout the world was estimated to be 164.7 million. 163.2 million occurred in developing countries, with 1.1 million deaths, and 1.5 million occurred in industrialized countries. More than half of the episodes and death affects children under 5 years of age. In comparing developing countries against industrialized countries, the median of isolates are S. flexneri (60% vs. 16%), S. sonnei (15% vs. 77%), S. dysenteriae (6% vs. 1%), and S. boydii (6% vs. 2%). The predominant serotype of S. flexneri in developing countries is 2a, followed by 1b, 3a, 4a, and 6, while in industrialized countries most isolates are S. flexneri 2a and unspecified type 2 strains. 10516787 PMC2557719

[2] von Seidlein L, Kim DR, Ali M, Lee H, Wang X, Thiem VD, et al A multicentre study of Shigella diarrhoea in six Asian countries: disease burden, clinical manifestations, and microbiology. PLoS Med. 2006;3(9):e353 Epub 2006/09/14. 10.1371/journal.pmed.0030353 ; PubMed Central PMCID: PMCPmc1564174. 16968124 PMC1564174

[3] Kotloff KL, Nataro JP, Blackwelder WC, Nasrin D, Farag TH, Panchalingam S, et al Burden and aetiology of diarrhoeal disease in infants and young children in developing countries (the Global Enteric Multicenter Study, GEMS): a prospective, case-control study. Lancet (London, England). 2013;382(9888):209–22. Epub 2013/05/18. 10.1016/s0140-6736(13)60844-2 . 23680352

[4] Collaborators GMaCoD. Global, regional, and national life expectancy, all-cause mortality, and cause-specific mortality for 249 causes of death, 1980–2015: a systematic analysis for the Global Burden of Disease Study 2015. Lancet (London, England). 2016;388(10053):1459–544. Epub 2016/10/14. 10.1016/s0140-6736(16)31012-1 ; PubMed Central PMCID: PMCPmc5388903. 27733281 PMC5388903

[5] DuPont HL, Levine MM, Hornick RB, Formal SB. Inoculum size in shigellosis and implications for expected mode of transmission. J Infect Dis. 1989;159(6):1126–8. Epub 1989/06/01. . 2656880 10.1093/infdis/159.6.1126

[6] Rossi O, Baker KS, Phalipon A, Weill FX, Citiulo F, Sansonetti P, et al Draft genomes of Shigella strains used by the STOPENTERICS consortium. Gut Pathog. 2015;7:14 Epub 2015/06/05. 10.1186/s13099-015-0061-5 ; PubMed Central PMCID: PMCPmc4454270. 26042182 PMC4454270

[7] Das SK, Ahmed S, Ferdous F, Farzana FD, Chisti MJ, Leung DT, et al Changing emergence of Shigella sero-groups in Bangladesh: observation from four different diarrheal disease hospitals. PloS one. 2013;8(4):e62029 10.1371/journal.pone.0062029 23658619 PMC3639224

[8] Mao Y, Cui E, Bao C, Liu Z, Chen S, Zhang J, et al Changing trends and serotype distribution of Shigella species in Beijing from 1994 to 2010. Gut pathogens. 2013;5(1):21. 23919811 10.1186/1757-4749-5-21 PMC3750644

[9] Qiu S, Xu X, Yang C, Wang J, Liang B, Li P, et al Shift in serotype distribution of Shigella species in China, 2003–2013. Clin Microbiol Infect. 2015;21(3):252.e5–8. Epub 2015/02/07. 10.1016/j.cmi.2014.10.019 . 25658535

[10] Talukder KA, Dutta DK, Safa A, Ansaruzzaman M, Hassan F, Alam K, et al Altering Trends in the Dominance of Shigella flexneri Serotypes and Emergence of Serologically AtypicalS. flexneri Strains in Dhaka, Bangladesh. Journal of clinical microbiology. 2001;39(10):3757–9. 10.1128/JCM.39.10.3757-3759.2001 11574611 PMC88427

[11] Toapanta FR, Bernal PJ, Kotloff KL, Levine MM, Sztein MB. T cell mediated immunity induced by the live-attenuated Shigella flexneri 2a vaccine candidate CVD 1208S in humans. Journal of translational medicine. 2018;16(1):61 10.1186/s12967-018-1439-1 29534721 PMC5851169

[12] Levine MM, Kotloff KL, Barry EM, Pasetti MF, Sztein MB. Clinical trials of Shigella vaccines: two steps forward and one step back on a long, hard road. Nature Reviews Microbiology. 2007;5(7):540 10.1038/nrmicro1662 17558427 PMC3771495

[13] Noriega FR, Liao FM, Maneval DR, Ren S, Formal SB, Levine MM. Strategy for cross-protection among Shigella flexneri serotypes. Infection and immunity. 1999;67(2):782–8. 9916090 10.1128/iai.67.2.782-788.1999 PMC96386

[14] Organization. WH. Generic protocol to estimate the burden of Shigella diarrhoea and dysenteric mortality. W H O/V and B/. 1999;99:26.

[15] Azmi IJ, Khajanchi BK, Akter F, Hasan TN, Shahnaij M, Akter M, et al Fluoroquinolone resistance mechanisms of Shigella flexneri isolated in Bangladesh. PLoS One. 2014;9(7):e102533 Epub 2014/07/17. 10.1371/journal.pone.0102533 ; PubMed Central PMCID: PMCPmc4100904. 25028972 PMC4100904

[16] Carlin NI, Lindberg AA, Bock K, Bundle DR. The Shigella flexneri O-antigenic polysaccharide chain. Nature of the biological repeating unit. Eur J Biochem. 1984;139(1):189–94. Epub 1984/02/15. . 6199198 10.1111/j.1432-1033.1984.tb07993.x

[17] Pryamukhina NS, Khomenko NA. Suggestion to supplement Shigella flexneri classification scheme with the subserovar Shigella flexneri 4c: phenotypic characteristics of strains. J Clin Microbiol. 1988;26(6):1147–9. Epub 1988/06/01. ; PubMed Central PMCID: PMCPmc266551. 3290245 10.1128/jcm.26.6.1147-1149.1988 PMC266551

[18] Luo X, Sun Q, Lan R, Wang J, Li Z, Xia S, et al Emergence of a novel Shigella flexneri serotype 1d in China. Diagn Microbiol Infect Dis. 2012;74(3):316–9. Epub 2012/08/04. 10.1016/j.diagmicrobio.2012.06.022 . 22858548

[19] Carlin NI, Lindberg AA. Monoclonal antibodies specific for Shigella flexneri lipopolysaccharides: clones binding to type I and type III:6,7,8 antigens, group 6 antigen, and a core epitope. Infect Immun. 1986;53(1):103–9. Epub 1986/07/01. ; PubMed Central PMCID: PMCPmc260082. 2424839 10.1128/iai.53.1.103-109.1986 PMC260082

[20] Carlin NI, Rahman M, Sack DA, Zaman A, Kay B, Lindberg AA. Use of monoclonal antibodies to type Shigella flexneri in Bangladesh. J Clin Microbiol. 1989;27(6):1163–6. Epub 1989/06/01. ; PubMed Central PMCID: PMCPmc267520. 2666435 10.1128/jcm.27.6.1163-1166.1989 PMC267520

[21] Kenne L, Lindberg B, Petersson K. Basic structure of the oligosaccharide repeating-unit of the Shigella flexneri O-antigens. Carbohydr Res. 1977;56(2):363–70. Epub 1977/07/01. . 332361 10.1016/s0008-6215(00)83357-1

[22] Knirel YA, Lan R, Senchenkova SN, Wang J, Shashkov AS, Wang Y, et al O-antigen structure of Shigella flexneri serotype Yv and effect of the lpt-O gene variation on phosphoethanolamine modification of S. flexneri O-antigens. Glycobiology. 2013;23(4):475–85. Epub 2013/01/04. 10.1093/glycob/cws222 . 23283000

[23] Liu B, Knirel YA, Feng L, Perepelov AV, Senchenkova SN, Wang Q, et al Structure and genetics of Shigella O antigens. FEMS Microbiol Rev. 2008;32(4):627–53. Epub 2008/04/22. 10.1111/j.1574-6976.2008.00114.x . 18422615

[24] Allison GE, Verma NK. Serotype-converting bacteriophages and O-antigen modification in Shigella flexneri. Trends Microbiol. 2000;8(1):17–23. Epub 2000/01/19. . 10637639 10.1016/s0966-842x(99)01646-7

[25] Simmons DA, Romanowska E. Structure and biology of Shigella flexneri O antigens. J Med Microbiol. 1987;23(4):289–302. Epub 1987/06/01. 10.1099/00222615-23-4-289 . 2438412

[26] Stagg RM, Tang SS, Carlin NIA, Talukder KA, Cam PD, Verma NK. A Novel Glucosyltransferase Involved in O-Antigen Modification of Shigella flexneri Serotype 1c. Journal of Bacteriology. 2009;191(21):6612–7. 10.1128/JB.00628-09 ; PubMed Central PMCID: PMCPmc2795301. 19717593 PMC2795301

[27] Carlin N, Lindberg A. Monoclonal antibodies specific for Shigella flexneri lipopolysaccharides: clones binding to type IV, V, and VI antigens, group 3, 4 antigen, and an epitope common to all Shigella flexneri and Shigella dysenteriae type 1 stains. Infection and immunity. 1987;55(6):1412–20. 2437036 10.1128/iai.55.6.1412-1420.1987 PMC260529

[28] Sun Q, Knirel YA, Lan R, Wang J, Senchenkova SN, Shashkov AS, et al Dissemination and serotype modification potential of pSFxv_2, an O-antigen PEtN modification plasmid in Shigella flexneri. Glycobiology. 2014;24(3):305–13. Epub 2014/01/01. 10.1093/glycob/cwt115 . 24379081

[29] Sun Q, Knirel YA, Lan R, Wang J, Senchenkova SN, Jin D, et al A novel plasmid-encoded serotype conversion mechanism through addition of phosphoethanolamine to the O-antigen of Shigella flexneri. PLoS One. 2012;7(9):e46095 Epub 2012/10/11. 10.1371/journal.pone.0046095 ; PubMed Central PMCID: PMCPmc3458804. 23049947 PMC3458804

[30] Sun Q, Lan R, Wang Y, Zhao A, Zhang S, Wang J, et al Development of a multiplex PCR assay targeting O-antigen modification genes for molecular serotyping of Shigella flexneri. J Clin Microbiol. 2011;49(11):3766–70. Epub 2011/09/02. 10.1128/JCM.01259-11 ; PubMed Central PMCID: PMCPmc3209073. 21880974 PMC3209073

[31] Ye C, Lan R, Xia S, Zhang J, Sun Q, Zhang S, et al Emergence of a new multidrug-resistant serotype X variant in an epidemic clone of Shigella flexneri. J Clin Microbiol. 2010;48(2):419–26. Epub 2009/12/04. 10.1128/JCM.00614-09 ; PubMed Central PMCID: PMCPmc2815595. 19955273 PMC2815595

[32] Connor TR, Barker CR, Baker KS, Weill FX, Talukder KA, Smith AM, et al Species-wide whole genome sequencing reveals historical global spread and recent local persistence in Shigella flexneri. Elife. 2015;4:e07335 Epub 2015/08/05. 10.7554/eLife.07335 ; PubMed Central PMCID: PMCPmc4522646. 26238191 PMC4522646

[33] Talukder KA, Islam MA, Dutta DK, Hassan F, Safa A, Nair GB, et al Phenotypic and genotypic characterization of serologically atypical strains of Shigella flexneri type 4 isolated in Dhaka, Bangladesh. J Clin Microbiol. 2002;40(7):2490–7. Epub 2002/06/29. 10.1128/JCM.40.7.2490-2497.2002 ; PubMed Central PMCID: PMCPmc120590. 12089268 PMC120590

[34] Talukder KA, Islam Z, Islam MA, Dutta DK, Safa A, Ansaruzzaman M, et al Phenotypic and genotypic characterization of provisional serotype Shigella flexneri 1c and clonal relationships with 1a and 1b strains isolated in Bangladesh. J Clin Microbiol. 2003;41(1):110–7. Epub 2003/01/09. 10.1128/JCM.41.1.110-117.2003 ; PubMed Central PMCID: PMCPmc149623. 12517835 PMC149623

[35] Qiu S, Wang Z, Chen C, Liu N, Jia L, Liu W, et al Emergence of a novel Shigella flexneri serotype 4s strain that evolved from a serotype X variant in China. J Clin Microbiol. 2011;49(3):1148–50. Epub 2010/12/24. 10.1128/JCM.01946-10 ; PubMed Central PMCID: PMCPmc3067715. 21177890 PMC3067715

[36] van der Ploeg CA, Rogé AD, Bordagorria XL, de Urquiza MT, Vinas MR, Pichel MG, et al AA479 antiserum: new reagent for the serotype characterization of atypical variants of Shigella flexneri. Revista Argentina de microbiologia. 2015;47(1):36–40. 10.1016/j.ram.2014.11.001 25735215

[37] Yang C, Li P, Zhang X, Ma Q, Cui X, Li H, et al Molecular characterization and analysis of high-level multidrug-resistance of Shigella flexneri serotype 4s strains from China. Sci Rep. 2016;6:29124 Epub 2016/07/05. 10.1038/srep29124 ; PubMed Central PMCID: PMCPmc4931504. 27374009 PMC4931504

[38] Organization WH. Programme for control of diarrhoeal disease In Manual for laboratory investigation of acute enteric infections 1987;CDD/93.3, rev. 1(World Health Organization, Geneva, Switzerland):9–20.

[39] Sun Q, Lan R, Wang J, Xia S, Wang Y, Wang Y, et al Identification and characterization of a novel Shigella flexneri serotype Yv in China. PloS one. 2013;8(7):e70238 10.1371/journal.pone.0070238 23936172 PMC3728103

[40] Kado C, amp, Liu S. Rapid procedure for detection and isolation of large and small plasmids. Journal of bacteriology. 1981;145(3):1365–73. 7009583 10.1128/jb.145.3.1365-1373.1981 PMC217141

[41] Haider K, Huq MI, Talukder KA, Ahmad QS. Electropherotyping of plasmid DNA of different serotypes of Shigella flexneri isolated in Bangladesh. Epidemiol Infect. 1989;102(3):421–8. Epub 1989/06/01. ; PubMed Central PMCID: PMCPmc2249461. 2661251 10.1017/s0950268800030132 PMC2249461

[42] Munshi MH, Sack DA, Haider K, Ahmed ZU, Rahaman MM, Morshed MG. Plasmid-mediated resistance to nalidixic acid in Shigella dysenteriae type 1. Lancet (London, England). 1987;2(8556):419–21. Epub 1987/08/22. . 2887725 10.1016/s0140-6736(87)90957-3

[43] Boisen N, Ruiz-Perez F, Scheutz F, Krogfelt KA, Nataro JP. High Prevalence of Serine Protease Autotransporter Cytotoxins among Strains of Enteroaggregative Escherichia coli. Am J Trop Med Hyg. 2009;80(2):294–301. ; PubMed Central PMCID: PMCPmc2660206. 19190229 PMC2660206

[44] Vargas M, Gascon J, Jimenez De Anta MT, Vila J. Prevalence of Shigella enterotoxins 1 and 2 among Shigella strains isolated from patients with traveler's diarrhea. J Clin Microbiol. 1999;37(11):3608–11. Epub 1999/10/19. ; PubMed Central PMCID: PMCPmc85705. 10523561 10.1128/jcm.37.11.3608-3611.1999 PMC85705

[45] Okada N, Sasakawa C, Tobe T, Talukder KA, Komatsu K, Yoshikawa M. Construction of a physical map of the chromosome of Shigella flexneri 2a and the direct assignment of nine virulence-associated loci identified by Tn5 insertions. Mol Microbiol. 1991;5(9):2171–80. Epub 1991/09/01. . 1662762 10.1111/j.1365-2958.1991.tb02147.x

[46] Talukder KA, Dutta DK, Albert MJ. Evaluation of pulsed-field gel electrophoresis for typing of Shigella dysenteriae type 1. J Med Microbiol. 1999;48(8):781–4. Epub 1999/08/18. 10.1099/00222615-48-8-781 . 10451002

[47] Edwards PR, and Ewing W. H. Identification of Enterobacteriaceae. Burgess Publishing Company, Minneapolis, Minn 1972:p. 126–31.

[48] Gentle A, Ashton PM, Dallman TJ, Jenkins C. Evaluation of Molecular Methods for Serotyping Shigella flexneri. J Clin Microbiol. 2016;54(6):1456–61. Epub 2016/03/18. 10.1128/JCM.03386-15 ; PubMed Central PMCID: PMCPmc4879286. 26984974 PMC4879286

